# Client Service Receipt Inventory as a standardised tool for measurement of socio-economic costs in the rare genetic disease population (CSRI-Ra)

**DOI:** 10.1038/s41598-021-03379-5

**Published:** 2021-12-13

**Authors:** Claudia C. Y. Chung, Jasmine L. F. Fung, Adrian C. Y. Lui, Marcus C. Y. Chan, Yvette N. C. Ng, Wilfred H. S. Wong, So Lun Lee, Martin Knapp, Brian H. Y. Chung

**Affiliations:** 1 Department of Paediatrics and Adolescent Medicine, LKS Faculty of Medicine, The University of Hong Kong, Pok Fu Lam, Hong Kong Special Administrative Region China; 2 Department of Paediatrics and Adolescent Medicine, Queen Mary Hospital, 1/F New Clinical Building, 102 Pokfulam Road, Pok Fu Lam, Hong Kong Special Administrative Region China; 3 The Duchess of Kent Children’s Hospital, Pok Fu Lam, Hong Kong Special Administrative Region China; 4 Care Policy and Evaluation Centre, Health and Social Care Policy, Department of Health Policy, London School of Economics and Political Science, Houghton Street, London, WC2A 2AE UK

**Keywords:** Outcomes research, Genetics

## Abstract

The measurement of costs is fundamental in healthcare decision-making, but it is often challenging. In particular, standardised methods have not been developed in the rare genetic disease population. A reliable and valid tool is critical for research to be locally meaningful yet internationally comparable. Herein, we sought to develop, contextualise, translate, and validate the Client Service Receipt Inventory for the RAre disease population (CSRI-Ra) to be used in cost-of-illness studies and economic evaluations for healthcare planning. Through expert panel discussions and focus group meetings involving 17 rare disease patients, carers, and healthcare and social care professionals from Hong Kong, we have developed the CSRI-Ra. Rounds of forward and backward translations were performed by bilingual researchers, and face validity and semantic equivalence were achieved through interviews and telephone communications with focus group participants and an additional of 13 healthcare professional and university students. Intra-class correlation coefficient (ICC) was used to assess criterion validity between CSRI-Ra and electronic patient record in a sample of 94 rare disease patients and carers, with overall ICC being 0.69 (95% CI 0.56–0.78), indicating moderate to good agreement. Following rounds of revision in the development, contextualisation, translation, and validation stages, the CSRI-Ra is ready for use in empirical research. The CSRI-Ra provides a sufficiently standardised yet adaptable method for collecting socio-economic data related to rare genetic diseases. This is important for near-term and long-term monitoring of the resource consequences of rare diseases, and it provides a tool for use in economic evaluations in the future, thereby helping to inform planning for efficient and effective healthcare. Adaptation of the CSRI-Ra to other populations would facilitate international research.

## Introduction

Rapid development of genomic medicine has led to increased public awareness of rare genetic diseases internationally, as it has vast amount of potential in screening, diagnosis, and precision medicine. Rare disease refers to conditions with rare occurrences in a population. According to the World Health Organization, rare disease affects less than five per 10,000 people in the European population^1^. These diseases are heterogeneous and individually rare, but collectively they affect 6% to 8% of the European population^2^. Patients with rare genetic diseases are often seen to be more complicated to treat and support due to their complex presentations and outcomes of multisystemic involvement. Most rare diseases have a genetic component and are usually chronically debilitating or life-threatening^3^. While the impact is often perceived as the immediate healthcare burden, the broader socio-economic consequences of rare diseases are extremely important for healthcare and related planning, yet are very challenging to estimate.

The healthcare and societal impacts that fall to individual patients, families, and communities as a consequence of rare diseases have an economic dimension. Costs of rare genetic diseases can be incurred at all levels of the society, both directly through healthcare expenditure and unpaid family or other carer support, and indirectly through reduced productivity and opportunities lost. Evidence on cost-of-illness studies and health economic evaluations in the rare disease population remains scarce, and most such studies focus on the direct healthcare costs associated with hospital services, specific genetic diagnostic tools, interventions, or treatments, and lack information on indirect and intangible costs in the broader societal perspective. Estimation of these wider socio-economic impacts and the underlying service and resource utilisation patterns associated with rare genetic diseases is important for near-term and long-term monitoring of resources, and for effective and efficient healthcare planning.

A valid and reliable approach to collect comprehensive healthcare and socio-economic data is required for research to be locally meaningful yet internationally comparable. However, standardised methods have not been developed for local or international research in the rare genetic disease population. One of the main 10-year global rare disease goals of the International Rare Diseases Research Consortium (IRDiRC) for 2017 to 2027 is to develop methodologies to evaluate the socio-economic impact of diagnoses and therapies on patients with rare diseases^4^, but there is currently no validated instrument to capture comprehensive healthcare and socio-economic data in this population. There is a need for a robust tool to collect comprehensive information on cost-related data that is both sensitive to the local context and sufficiently standardised to balance local relevance with international generalisability.

One of the most widely used resource-use measurement tools is the Client Service Receipt Inventory (CSRI)^5^. The CSRI is a comprehensive research tool developed in the United Kingdom in the 1980s to collect information on cost-related data^6,7^. Taking approximately 20 minutes to complete (although shorter versions have also been used for some specific contexts), its major purpose is to describe and measure service utilisation patterns as a basis for estimating associated costs across healthcare, social care and community settings. There are different versions of the CSRI, adapted to cater for different disease areas, age groups, healthcare systems, languages, and modes of administration. The CSRI has been used in over 700 studies in different populations, including the United Kingdom, Germany, Denmark, Spain, Italy, the Netherlands, Australia, New Zealand, and Brazil. The overall structure and content of the CSRIs have remained similar, containing five main sections: Background information, Household and carer support, Healthcare service and resource utilisation, Community support, and Education and employment. Previous studies have shown that CSRI is a reliable and valid tool for collection and estimation of economic data^8–10^. The development and adaptation of the CSRI in the rare genetic disease population for the first time in any language and jurisdiction would allow the collection of comprehensive service and resource utilisation data as a platform for cost estimation, and would maintain sufficient standardisation for international adoption. It would also provide a tool for use in economic evaluations in the future, including cost–benefit and cost-effectiveness studies, with important implications on treatment and care service development.

In recent years, rare genetic disease has gained greater public awareness internationally. Yet, the social-economic burden of rare genetic diseases has never been reported in the Asia Pacific region. In the 7.5 million population in Hong Kong, it was estimated that one in 67 people has one or more rare disease, representing 1.5% of the population^11^. Hong Kong has a two-tiered healthcare system in which the public and private healthcare systems complement each other^12,13^. The public healthcare system, managed by the Hospital Authority (HA), acts as a safety net for the general population and the rare disease community, providing hospital and related services at the secondary and tertiary level^13^; in particular, over 90% of the inpatient admissions take place in the public hospitals in Hong Kong^14^. All public healthcare records within HA are available in the Clinical Data Analysis and Reporting System (CDARS) in an unlisted and anonymous manner. In 2015–2016, the inpatient cost of rare diseases was estimated to be HK$1,594,339,530 (US$204,402,504) in Hong Kong using the CDARS database, equivalent to 4.3% of the total inpatient expenditure by HA, highlighting the disparity between rare disease prevalence and associated inpatient burden^11^. In contrast, the private sector acts as a major provider for primary services, complementing the public sector by providing more personalised and accessible choices for those who can afford and are willing to pay^13^. However, the broader socio-economic burden of rare genetic diseases is yet to be estimated, due to the lack of a standardised tool for the collection of cost-related data beyond the public health system perspective, which hinders the assessment of the true burden of rare genetic diseases, and in turn makes it harder to reach decisions about the best use of scarce societal resources more broadly. The current study sought to (i) develop and contextualise the CSRI to fit the rare genetic disease population (CSRI-Ra) in Hong Kong for the first time to collect comprehensive healthcare and socio-economic data, (ii) translate the CSRI-Ra into traditional Chinese, and (iii) validate the CSRI-Ra in Chinese Hong Kong rare disease patients and carers. Translation and validation of the CSRI-Ra are especially important in this linguistically different population as translation errors could misrepresent its original purpose and compromise its reliability and validity.

## Methods

This study consisted of three stages: (i) development and contextualise of the CSRI-Ra, (ii) translation, and (iii) validation. The good-practice checklist and flow diagram for resource-use data collection methodology funded by the United Kingdom Health Technology Assessment (UK HTA) program were used as reference^5,15^. Ethics approval was granted by the Institutional Review Board, the University of Hong Kong/Hospital Authority Hong Kong West Cluster (UW 19-609). The study was carried out in accordance to the principles set out in the Declaration of Helsinki.

### Development and contextualisation of the CSRI-Ra

Two focus groups meetings were conducted to develop and contextualise the CSRI-Ra specifically for the rare genetic disease population in Hong Kong. The first focus group meeting consisted of rare disease patients and family members/informal carers, focusing on service and resource needs and utilisation for the rare genetic disease population in Hong Kong. Patients and carers of patients with rare diseases were recruited via Rare Disease Hong Kong (RDHK), the first and largest rare disease patient organisation in Hong Kong. The purpose of the first focus group was to collect data specific to the rare disease population, for the development of the CSRI-Ra. The second focus group brought together healthcare and social care professionals who are experienced in working with patients with rare genetic diseases. An invitation email was sent to a randomly selected list of special schools and non-governmental organisations (NGO) in Hong Kong, indicating the study’s objective and scope, targeting any allied health workers and staff who have experience in working with patients with rare genetic diseases. This focus group was to understand and contextualise the tool to be adapted in the local context, taking into account the healthcare and social care structure or system in Hong Kong, and factors relating to patients’ access to specific services and resources. All participants were informed of the study's objectives and data confidentiality standards, and written informed consent was obtained from all participants.

The focus group meetings were conducted in Cantonese Chinese and were video- and audio-recorded. Factors revolved around the CSRI-Ra were discussed in each focus group meeting, including the content, structure, format, retrospective period, data collection approach (self-completed inventory, face-to-face interview, or telephone interview). At each session, two moderators and two observers were present to lead the discussion and to observe and make field notes. A debriefing session was conducted immediately after each meeting to consolidate comments, suggestions, criticisms, and observations to develop and contextualise the CSRI-Ra. Participation was anonymised to preserve confidentiality.

Data collected from the focus group meetings were analysed using Braun and Clarke’s framework for thematic analysis^16^. The six phases of thematic analysis described by Nowell et al. was used as reference for conducting the analysis^17^. Combined inductive and deductive approach was employed to allow themes to be generated based on pre-existing CSRIs and literature, while leaving space to discover other novel aspects of the participants’ experience. Open coding and broad-brush coding were used, and the comments and viewpoints were grouped into emerging semantic themes by two independent researchers. Discrepancies were identified and resolved.

All identified themes, subthemes, and variables were further grouped into the usual five key sections of the CSRIs: Background information, Household and carer support, Healthcare service and resource utilisation, Community support, and Education and employment. The original published CSRIs in other healthcare areas available from the Personal Social Services Research Unit (PSSRU), University of Kent, were used as frameworks for development of the CSRI-Ra^7,18–23^. The Database of Instruments for Resource-Use Management (DIRUM), an open-access database of resource-use questionnaires for use by health economists and a repository of methodological papers related to resource use and cost measurement, was also used as a reference for the development of the CSRI-Ra^24,25^.

An expert panel including the clinical geneticist, other medical subspecialists, genetic counsellor, health economist, and developer of the original CSRI, was formed to discuss on the overall structure of the CSRI-Ra, and to ensure that the reliability and accuracy of constructs were achieved.

### Translation of the CSRI-Ra

After the development of the CSRI-Ra for the rare disease population and the contextualisation to the Hong Kong context, the CSRI-Ra was forward translated to traditional Chinese by two bilingual translators, then backward translated to English by another two independent bilingual translators. The aim of this process was not a word-to-word translation, but rather a translation that could accurately reflect the research context. Discrepancies were identified and resolved accordingly. Quotes from focus group meetings, interviews, and expert panel discussions that were relevant to the identified themes were also translated into English and reported in the “Results” section.

### Validation of the CSRI-Ra

There were two aims of the validation process: (i) to achieve face validity and semantic equivalence within individuals, and (ii) to test the reliability, validity, and accuracy of the CSRI-Ra using statistical analyses.

To achieve face validity and semantic equivalence within individuals, the translated CSRI-Ra (both English and traditional Chinese versions) were sent back to all focus group participants (rare genetic disease patients, family members, carers, healthcare and social care professionals). Email and telephone communication were made with the participants to ensure that their comments and thoughts raised in the focus group meetings were reflected. This also ensured that the translated CSRI-Ra could be understood by the rare disease community. In addition, face-to-face interviews with three other allied health professionals (one senior nurse and two medical social workers) who were not involved in the focus group meetings were conducted to address the content and language of the CSRI-Ra. Face-to-face discussion with bilingual university students (not involved in the focus group meeting) was also performed to ensure that the CSRI-Ra could be understood by laypersons in the general population. Another expert panel discussion was conducted to reach consensus.

Reliability, validity, and accuracy of the CSRI-Ra were assessed using a sample of Hong Kong Chinese rare genetic disease patients and carers, recruited via RDHK and other social media platforms on a strictly voluntary basis. Informed consent was obtained from all participants.

For the purpose of assessing the reliability between the English and Chinese versions, bilingual patients and carers completed both the English and Chinese versions of the CSRI-Ra, with a time gap of approximately one month. Intra-class correlation coefficient (ICC) was used to estimate the variation of the data collected by the English and Chinese versions. A two-way random-effect model based on single ratings and absolute agreement assessed the alternate-form reliability of the two versions. Mean estimations along with 95% confidence intervals (CIs) were reported for each ICC. ICC was measured on a scale of 0 to 1, with ICC values less than 0.5 indicative of poor reliability, between 0.5 and 0.75 indicative of moderate reliability, values between 0.75 and 0.9 indicative of good reliability, and values greater than 0.9 indicative of excellent reliability^26–28^. Sample size calculation revealed that eight participants were required to demonstrate the significant association between the English-Chinese translation.

For the purpose of assessing the validity and accuracy of CSRI-Ra, the healthcare utilisation record from the electronic patient record (ePR) was retrieved for comparison. In Hong Kong, all public healthcare records under the management of HA are recorded in the ePR. Patients and carers recruited in this validation stage were informed of the objectives and data confidentiality standards, and gave informed consent for the research team to access to their ePR. Participation was completely voluntary. Self-/proxy-reported healthcare service utilisation record collected from the CSRI-Ra was compared with the actual healthcare service utilisation record collected from the ePR. Number of inpatient, outpatient, and accident and emergency (A&E) admissions were compared. The retrospective period over which data sought was the same in both the CSRI-Ra and ePR (six months retrospective period); data collection via ePR was dated back six months from the date of CSRI-Ra completion. Data collected were deidentified and were kept strictly confidential. Criterion validity between CSRI-Ra and ePR was analysed using ICC with two-way random-effect model. It was hypothesised that highest agreement would be observed in the area of inpatient admission as more than 90% of inpatient admissions take place in the public hospitals in Hong Kong^14^. On the contrary, it was hypothesised that less agreement would be observed between the CSRI-Ra and ePR in the area of outpatient visits, as private healthcare sector plays a major role in outpatient service support in Hong Kong, meaning that some of the outpatient service utilisation record would not be captured by the ePR; it was also expected that A&E service may not be as significant in this chronic illness population.

All statistical analyses were conducted using SPSS version 26.0 (IBM SPSS Statistics).

### Ethics declaration

Ethics approval was granted by the Institutional Review Board, the University of Hong Kong/Hospital Authority Hong Kong West Cluster (UW 19-609). Participants recruited in this study were informed of the objectives and data confidentiality standards. Written informed consent was obtained from all participants as required by the IRB. The study adheres to the principles set out in the Declaration of Helsinki.

## Results

### Development and contextualisation of CSRI-Ra

#### Focus group meetings

A total of 17 participants were recruited to participate in the focus group meetings: five males (29.4%) and 12 females (70.6%). Eleven (64.7%) participants had not completed any rare disease-related survey before, and 15 (88.2%) participants had not completed any service or resource utilisation survey before.

The first focus group meeting consisted of a total of eight rare disease patients and family members/informal carers of rare disease patients, covering six unique rare genetic diseases. Demographic characteristics of the first focus group participants are summarised in Table 1. The six rare genetic diseases are achondroplasia, Marfan syndrome, mucopolysaccharidosis type 6, Pompe disease, tuberous sclerosis, and Williams syndrome. These six rare genetic diseases were considered to be a good representation of the rare genetic disease population in Hong Kong by the expert panel, as they cover different types of impairment, including physical disability, intellectual disability, mental health problems, and visual impairment, reflecting how variable the rare genetic disease population could be. In addition, these six rare diseases also reflected the availability and unavailability, and the need of expensive medications, treatments, and resources to manage the disease.

**Table 1:** Characteristics of the eight rare disease patients or carers in the first focus group meeting.

Characteristics	Number (%)
**Role**	
Rare disease patient	2 (25.0)
Family member/informal carer of patient with rare disease	6 (75.0)
**Gender**	
Female	5 (62.5)
Male	3 (37.5)
**Age group**	
18–25	0 (0.0)
26–30	1 (12.5)
31–35	0 (22.2)
36–40	2 (25.0)
41–45	1 (12.5)
> 45	4 (50.0)
**Education level**	
Primary or below	0 (0.0)
Secondary	0 (0.0)
Post-secondary/associate degree or equivalent	1 (12.5)
Bachelor’s degree	2 (25.0)
Master’s/doctoral degree	5 (62.5)
**Rare disease**	
Achondroplasia	1 (12.5)
Marfan syndrome	2 (25.0)
Mucopolysaccharidosis type 6	2 (25.0)
Pompe disease	1 (12.5)
Tuberous sclerosis	1 (12.5)
Williams syndrome	1 (12.5)
**Previous experience in completing questionnaires for research purposes**	
Questionnaires related to rare diseases	6 (75.0)
Questionnaires related to service use/resource use	2 (25.0)

The second focus group meeting consisted of nine healthcare and social care professionals who are experienced in working with patients with rare genetic diseases. Their characteristics are summarised in Table 2. The average length of experience related to rare diseases was 12.0 years (standard deviation (SD): 6.8; range: 5–25). Their targeted service patient groups included patients with physical disability, intellectual disability, mental health problems, visual impairment, hearing impairment, and others; with age spanned from infants (< 1 year) to older adults (≥ 65 years).

**Table 2:** Characteristics of the nine professional participants in the second focus group meeting.

Characteristics	Number (%)
**Role**	
Nurse (based in special school)	1 (11.1)
Social worker	2 (22.2)
Special school principal/teacher	3 (33.3)
Manager/coordinator/staff in non-governmental organisation	3 (33.3)
**Gender**	
Female	7 (77.8)
Male	2 (22.2)
**Age group**	
18–25	0 (0.0)
26–30	2 (22.2)
31–35	0 (22.2)
36–40	3 (33.3)
41–45	1 (11.1)
> 45	3 (33.3)
**Education level**	
Primary or below	0 (0.0)
Secondary	0 (0.0)
Post-secondary/associate degree or equivalent	2 (22.2)
Bachelor’s degree	3 (33.3)
Master’s/doctoral degree	4 (44.4)
**Length of experience related to rare disease (years)**	
< 5	0 (0.0)
5–9	4 (44.4)
10–14	2 (22.2)
15–19	1 (11.1)
≥ 20	2 (22.2)
Average years of experience (SD)	12.0 (6.8)
**Target patients type**	
Physical disability	6 (66.7)
Intellectual disability	9 (100.0)
Psychological/mental problems	3 (33.3)
Visual impairment	6 (66.7)
Hearing impairment	5 (55.6)
Others	1 (11.1)
**Target patient group (years of age)**	
Infant (< 1)	1 (11.1)
Toddler (1–2)	1 (11.1)
Child (3–12)	5 (55.6)
Adolescent (13–18)	6 (66.7)
Adults (> 18)	5 (55.6)
Older adults (≥ 65)	3 (33.3)
**Previous experience in completing questionnaires for research purposes**	
Questionnaires related to rare diseases	0 (0.0)
Questionnaires related to service use/resource use	0 (0.0)

*SD* Standard deviation

#### Form of the instrument

One of the major discrepancies of this CSRI-Ra compared with the previous CSRIs was the availability of both the self-completed version (patient’s version) and the proxy-completed version (carer’s version). With rare genetic diseases’ heterogeneity and variable severity, the need to have both a patient’s and a carer’s version was consistently highlighted in the focus group meetings. Patients who can understand and complete the CSRI-Ra should complete the patient’s version in a self-completion format. For those patients who are unable to complete the CSRI-Ra by themselves, including but not limited to patients who are cognitively or otherwise unable to report their own utilisation record, the patient’s main unpaid carer (e.g., parent, spouse, child, etc.) should act as a proxy and complete the CSRI-Ra carer’s version. There is no strict rule or assessment for deciding which version to be employed.

The retrospective period over which data were sought was discussed in the focus group meetings. With reference to the previous CSRIs and other published cost-of-illness studies, and with the necessity to capture services that are infrequently used (i.e., inpatient admission), a retrospective period of six months was agreed, to balance the tool’s comprehensiveness and the possibility of recall error.

#### CSRI-Ra content: thematic analysis

All emerging themes identified from the focus group meetings were grouped into the usual five key sections in the CSRIs: Background information, Household and carer support, Healthcare service and resource utilisation, Community support, and Education and employment (Table 3). Example quotes were also provided to aid the understanding of the key variables included in the CSRI-Ra.

**Table 3:** Key sections and variables of the CSRI-Ra supported by key themes and example quotes.

CSRI-Ra section	CSRI-Ra key variables	Key themes	Example quotes
Background information	Date of questionnaire completion, gender, date of birth, ethnicity, marital status, rare disease, genetic change, year of diagnosis, number of other family member(s) with rare disease *Carer’s (proxy-completed) version: background information of the carer (the person who complete the questionnaire) will also be asked	Rare disease characteristics Patient’s demographics Carer’s demographics	“My brother has the same rare disease as I do” “I am not brave enough to get married. I wouldn’t even dare to think about that.” [Rare disease patient] “It is very important to have both the patient-completed and carer-completed versions as rare diseases can be very heterogeneous. They can affect both children and adults, some conditions are very severe while some can be very mild where patients can function normally and go to school or work”
Household and carer support	Type of residence, living situation (alone, with parents, etc.), home modifications, in-home care by paid and/or unpaid carer(s), number of paid carer(s) hired, number of working hours reduced or days absent for unpaid carer(s), impact on unpaid carer(s)’ employment, household income	Impact on daily living at home Paid care Informal care Financial burden	“My parents helped to take care of my son [rare disease patient]. They are his main carers, as we both need to work” “We need to hire domestic helpers. In fact it is very difficult to hire domestic helpers. They need to know all the professional caring skills, and it is often really difficult for the domestic helpers too. Therefore it is not uncommon to see them quitting, and we basically have to keep hiring new domestic helpers. (…) In fact this is such a heavy burden for a lot of the families, such as that in our case. It’s the financial and psychological burden that the patient and the carers face…” “A lot of modifications were made at home, such as having a hoist… and our toilet was specially designed for the disabled; we have many handrails…”
Community support	Social security support received by patient and family member(s) of the same household, type and cost of transportation utilised to get to healthcare and community service/resource providers, centre services provided by non-governmental organisations, patient support group	Community support Transportation	“In fact expenditure on transportation is really high… yes for us it would be great if we can take bus and minibus, but for other wheelchair-bounded patients, they will need to use rehabus or taxi. (…) This also impacts their living style. Some prefer to stay home all the time due to very high transportation fee, and they may even skip hospital follow-up because of this” “Patient support groups are extremely important for patients and carers. They get to socialise with each other, and they can support and teach each other new knowledge about management skills and updates of the disease, etc. It is something invaluable that we should raise more awareness about”
Healthcare service and resource utilisation	A&E visits, hospital inpatient days, outpatient attendances, day care attendances, allied health visits, surgeries/medical procedures/treatment, “Community Medical Service” program provided by the Hospital Authority, alternative medicine, medications, medical devices/consumables, healthcare/community services utilised by other family members, out-of-pocket healthcare expenditure	Patient’s healthcare needs to maintain physical and psychological health Out-of-pocket expenditure	“My husband’s rare disease requires extremely high expenditure on surgery and related resources. At his fourth surgery, he had to stay in the intensive care unit for 49 days, and the entire inpatient experience for that single admission lasted for more than 80 days” “Public and private healthcare services target different parts of the disease, even when it is the same service… for example PT OT ST [physiotherapy, occupational therapy, speech therapy]… and especially when there is a very long waiting list for public healthcare services provided by the Hospital Authority, we would rather pay out-of-pocket and pay more to use private services” “In fact my brother has already undergone the surgery for his rare disease condition. He still requires a lot of different medical devices to maintain his daily living. For example, he requires 2 to 3 ventilators, and each of them costs approximately 100 thousand Hong Kong dollars, and we still need to pay for the maintenance fee, not to mention the costs of other consumables… These pose a huge burden to us patients and carers who are among the grassroots of the society…”
Education and employment	Education level, education/employment status, type of school, visits to healthcare and social care professionals at school, whether the rare disease has affected learning/work, related problems at school/work, frequency of problems, days of school/work absent, source of income, working days and hours, impact on employment, education/employment status before diagnosis *Carer’s (proxy-completed) version: employment status of the carer (the person who complete the questionnaire) will also be asked	Opportunities and productivity loss Problems encountered at school/work	“Some rare disease patients get really low conduct marks in mainstream school as they are always absent from classes for medical follow-up.” “We both [patient and patient’s wife] retired early… because of the medical report… How would you make your life choices when you know that there’s a chance that you guys will be parted forever…? at the age of mid 40 s…? (teared) (…) We have both obtained Master’s degree, (…) we have studied for so many years, and we are now only at our mid 40 s… you could probably imagine how significant was the “lost” to us” We have saved all our annual leaves for our son since he will probably need spontaneous surgeries. Sometimes I will also take half day off to bring him to medical follow-ups, such as occupational therapy and physiotherapy. I basically go to every single session with him”

This table is based on the patient’s version of the CSRI-Ra. The patient’s and carer’s versions are almost identical, with minor differences in section orders.

*A&E *accident and emergency, *CSRI-Ra* Client Service Receipt Inventory for RAre genetic disease population.

##### Background information

The background section collects information on demographic and rare disease characteristics, and is generally the same as previous CSRIs. A variety of categorised variables, including gender, date of birth, ethnicity, marital status, rare genetic disease, and number of other family member(s) with rare genetic disease, made up the first section of the CSRI-Ra.

##### Household and carer support

Accommodation is an important element for economic studies of the rare genetic disease population, mainly because of the additional support required that could be reflected in the patient’s living situation (alone, with family members, or with paid caregivers). In addition, the CSRI-Ra collects information on home modifications made (i.e., handrails, patient hoist, shower chair, etc.) as a result of the patient’s condition. These potentially comprise significant economic costs for rare genetic diseases. On the other hand, most rare genetic diseases are chronically debilitating or life-threatening, and it is likely that patients with rare diseases would require a lot of paid or unpaid carer support. The professional background (e.g. domestic helper, hourly-paid home care assistant) and the number of paid carer(s) hired were collected in the CSRI-Ra. If patients were taken care of by unpaid carer(s), their relationship with the patient, the number of working hours reduced per day, and days absent from work were collected. The number of hours the paid/unpaid carer(s) spent on taking care of the rare disease patient was also collected.

##### Community support

A range of social security supports provided by the government were included, including Comprehensive Social Security Assistance (CSSA) and Social Security Allowance (SSA) Schemes. Centre services provided by non-governmental organisations, and whether the patient is a member of any patient support group, were also requested. In addition, participants were also asked about their usage of, and money spent on transportation to utilise healthcare and community services and to purchase resources.

##### Healthcare service and resource utilisation

An individual’s use of any medical services, procedures, and medications from the public and private healthcare sectors were requested, and together were considered to make up a comprehensive profile of services and resources available to the patient population. This included healthcare services (A&E, inpatient, outpatient, day care, allied health, and “Community Medical Service” program), medical procedures/surgeries, alternative medicine (e.g. Chinese medicine, acupuncture, massage therapy), medications, and medical devices/consumables (e.g. wheelchair, ventilator, hearing aid, disinfectant). Where applicable, the frequency, duration, dosage and whether it was provided by the public/private sector, etc. were requested in the CSRI-Ra.

##### Education and employment

This section is slightly different for the patient’s and carer’s versions of the CSRI-Ra. This section aims to collect information on patient’s education/employment status. For the carer’s version, the carer’s education/employment status was also collected. This section is a fundamental part to estimate the indirect costs of rare genetic disease, such as the number of days absent from work due to the rare disease or due to taking care of the rare disease patient, reflecting the productivity loss associated with rare genetic diseases.

The education/employment status was divided into seven categories, including student, full-time employment, part-time employment, housewife/househusband, retired, unemployed, and others. For students, the type of school or course attending, such as mainstream school, special school, or hospital school, etc., was requested. Participants were also asked about whether the patient has visited any allied health professionals at school, including school nurse, social worker, physiotherapist, speech therapist, special educational needs coordinator, etc. For individuals who are currently employed, the full time/part time position was requested. Impact on employment opportunity was also collected, which may include early retirement, changed from full-time to part-time job, unemployment, change in working hours, etc. For patients who are either studying or working, they were asked about whether their rare disease condition has affected their learning/work and its associated reasons, reasons may include health condition restriction, being tired, feeling worried or anxious, inability to concentrate, leaving school/work early or arriving late due to the need to attend medical appointments, etc. The number of days absent from school/work due to their rare disease condition was also requested. Education/employment status prior to being diagnosed with their rare disease was asked in order to examine the change in status due to the condition.

In the carer’s version, other than patient’s education/employment status, data on the carer’s (the proxy who complete the CSRI-Ra) education/employment was also collected. This would allow the estimation of the carer’s productivity loss due to taking care of a rare genetic disease patient.

### Translation and validation of CSRI-Ra

#### Reliability between English and Chinese versions of the CSRI-Ra

Through forward and backward translations, the English and Chinese versions of the CSRI-Ra had been developed. In the light of recommendations on content, terminology and language through email and telephone communication with all focus group participants, face-to-face discussion with ten bilingual university students, and more formally through face-to-face interviews with one senior nurse and two medical social workers, the tool’s face validity and semantic equivalence were achieved. The overall ICC was excellent being 0.91 (95% CI 0.89–0.92). Details of the translation process and the results on alternate-form reliability are included in the Supplementary Document.

#### Agreement between CSRI-Ra and electronic patient record

A total of 94 patients (n = 54) and carers (n = 40) provided informed consent for the research team to access to their ePR. There were 56 (59.6%) females and 38 (40.4%) males. The mean age of the patients (self-completed version) and carers (proxy-completed version) were 40.8 years (SD: 17.5) and 46.8 years (SD: 15.3), respectively. Majority of the participants’ highest education level was Secondary level (46.8%), followed by Bachelor’s Degree level (18.1%), Post-Secondary/Associate Degree or equivalent (14.9%), Master’s/Doctoral level (13.8%), and Primary level or below (4.3%). Two participants did not provide their highest education level. A total of 45 distinct rare diseases were covered.

The overall ICC was between moderate and good (ICC 0.69; 95% CI 0.56–0.78), demonstrating reasonable criterion (concurrent) validity (Table 4). The ICC for both the patient’s (self-completed) and carer’s (proxy-completed) versions were between moderate and good, with the ICC of the patient’s version being 0.67 (95% CI 0.50–0.80), and carer’s version being 0.70 (95% CI 0.50–0.83).

**Table 4:** External validity between CSRI-Ra versions and ePR using intra-class correlation coefficient.

	CSRI-Ra version		
	Overall (both self-completed and proxy-completed versions)	Self-completed version (patient’s version)	Proxy-completed version (carer’s version)
Overall service utilisation (IP, OP, A&E)	0.69 (95% CI 0.56–0.78)	0.67 (95% CI 0.50–0.80)	0.70 (95% CI 0.50–0.83)
IP admission	0.81 (95% CI 0.73–0.87)	0.81 (95% CI 0.70–0.89)	0.79 (95%CI 0.64–0.89)
OP visits	0.60 (95% CI 0.45–0.71)	0.46 (95% CI 0.22–0.65)	0.67 (95% CI 0.46–0.81)
A&E visits	0.58 (95% CI 0.42–0.70)	0.66 (95% CI 0.48–0.79)	0.33 (95% CI 0.03–0.58)

*A&E* accident and emergency, *CI* confidence interval, *CSRI-Ra* Client Service Receipt Inventory for RAre genetic disease population, *ePR* electronic patient record, *IP* inpatient, *OP* outpatient.

Subgroup analysis on item performance revealed convergent validity and discriminant validity of the tool. As hypothesised, better agreement between CSRI-Ra and ePR was observed in inpatient admissions (ICC 0.81; 95C CI 0.73–0.87), regardless of whether the CSRI-Ra was self-completed (ICC 0.81; 95% CI 0.70–0.89) or proxy-completed (ICC 0.79; 95% CI 0.64–0.89). In contrast, less agreement was observed in outpatient visits (ICC 0.60; 95% CI 0.45–0.71) and A&E visits (ICC 0.58; 95% CI 0.42–0.70).

The main findings regarding service utilisation and costs for this sample are beyond the scope of this article and are to be reported elsewhere.

## Discussion

After rounds of revision in the development, contextualisation, translation, and validation stages, the CSRI-Ra is now ready for use in empirical research in the rare genetic disease population in Hong Kong, as well as for exploratory use elsewhere. This is, we believe, the first validated resource-use instrument for the rare genetic disease population internationally. The CSRI-Ra is easy to understand and complete, takes only 20–30 minutes to complete, can be completed by the patient or carer, is available in both English and Chinese, and is shown to be a reliable and valid tool for the collection of comprehensive resource-use information. The CSRI-Ra is important for monitoring healthcare utilisation patterns and estimating economic costs. This study provides evidence to suggest that the CSRI-Ra may have value as a standard resource-use questionnaire in the rare genetic disease population. The CSRI-Ra and manual are available to researchers from https://paed.hku.hk/e-form/csri-ra-registration-form.asp upon reasonable request.

Patient/proxy-reported questionnaires offer a structured means of gathering information simultaneously on the use of a broad range of health, social care and other services, out-of-pocket payments, and informal support activities that may not be collected by other methods. They therefore have the advantage of capturing data on resource items that may be included in ePR and other administrative systems, but not linked in ways that show the whole service picture for any individual, as well as data on resource items falling outside these systems because they are purchased privately by patients and families, or are less tangible, such as informal care and support. However, in doing so, the CSRI-Ra and the previous CSRIs rely on the participant’s recall about services and supports used during the specified retrospective time period. There is considerable debate as to whether the “reality” could be accurately reflected due to recall bias. The number of different recall periods should be kept to a minimum in the questionnaire, as switching between different durations of recall in different questions could be challenging for respondents. The recommended recall period was indicated at a maximum of six months, allowing services that are considerably “rarer” (i.e., infrequently used, yet possibly quite high cost) to be captured, yet balancing participants’ recall ability^7^. In this study, a recall period of six months was agreed by patients and carers from the rare disease community, and by professionals and experts from different healthcare and social care areas. Our study demonstrated that data collected based on participant’s recall is comparable to data collected using ePR, with an overall ICC of 0.69 (95% CI 0.56–0.78), indicating moderate to good agreement, achieving reasonable criterion (concurrent) validity. This is consistent with previously published studies that compared CSRI and medical records in collecting resource use data, demonstrating satisfactory level of concordance^8–10^. As hypothesised, better agreement was observed in the area of inpatient admission, compared with that of outpatient and A&E visits. This could potentially be explained by the health system structure in Hong Kong, in which over 90% of inpatient admissions take place in the public hospitals under the management of HA, and therefore could be recorded in the ePR^14^. On the contrary, approximately 68% of outpatient services were supported by the private healthcare sector, whereby service utilisation record would not be available from the ePR^29^. The CSRI-Ra on the other hand, allows service use data in the private healthcare system, and data associated with indirect costs (i.e., travel costs, productivity loss, etc.) to be captured, allowing a more comprehensive perspective in estimating costs of rare genetic diseases. A direct source of information that relies on participants’ recall is often considered to be the strongest level of evidence for service use collection and assessment^30^.

The CSRI-Ra is sufficiently standardised by adapting the existing CSRI, yet maintaining local relevance by taking into account the Hong Kong healthcare and social care systems and structures. To increase generalisability to other populations and contexts, mode of administration and age-appropriateness of instrument are two important factors to be considered^25^. The current CSRI-Ra offers two modes of administration, self-completed and proxy-completed versions, and allows data collection in a wide range of patient age-groups. This is the first CSRI version that offers both patient-completed and proxy-completed versions. Rare genetic diseases are heterogeneous; they can affect any age groups, be of variable severity, and can involve different organs and systems. The availability of both patient-administered and proxy-administered versions ensures that rare disease patients of different age groups and different levels of impairment can be captured. Data can also be collected for rare genetic disease patients in other populations and healthcare systems by the patient-/proxy-completed versions. However, slight contextualisation will need to be performed for the tool to capture nuances of healthcare systems and social support structures in other populations.

The availability of an electronic version of the CSRI-Ra was recommended by a few participants during the validation stage. There are obvious benefits of developing an electronic version, including easier distribution, the function of skipping from question to question, shortening of the questionnaire based on the answer provided (i.e., the whole section can be skipped), easier participation (i.e., respondents can choose when to complete the questions such as when commuting to work), sharing of the tool via social media platforms, etc. However, participants without access to online means or those unfamiliar with electronic devices would not then be recruited into studies. Issues with confidentiality, data-sharing, and data security would also need to be addressed. In the future, the development of an electronic version can be considered, but this should not replace the paper version of the CSRI-Ra.

Some limitations should be acknowledged. First, the sample size for validation of the English-Chinese translation is small. The number of bilingual rare genetic disease patients and carers who have not participated in the development and contextualisation stages is relatively limited in Hong Kong. The excellent alternate-form reliability might be overestimated due to the small sample size, though sample size calculation revealed that eight subjects were sufficient to demonstrate the significant association between the translations. Second, criterion validity between the CSRI-Ra and ePR considered only inpatient, outpatient, and A&E services in the public healthcare system. Level of consistency was not assessed in other areas including private healthcare services, community services, employment status, etc. With the inaccessibility of databases for research purposes (i.e. organisations’ own internal affairs), unavailability of data related to indirect costs (i.e. waiting time, travel costs, etc.), ethical and confidentiality considerations, and issues with completeness, reliability, and validity of databases, criterion validity analysis could only be performed in certain public healthcare areas^30^. Yet, with the fairly well demonstrated agreement between recall data and ePR data, it was anticipated that other areas reported in the CSRI-Ra could reflect the socio-economic costs associated with rare diseases.

The CSRI-Ra, when used alone, or when used to complement other methods such as routine medical records from ePR, provides valuable data for the estimation of rare genetic disease costs, and may inform healthcare planning. The instrument can therefore also be used to collect cost data for use in trials and other evaluations—alongside clinical outcome data—so as to explore the cost-effectiveness of alternative treatment or care strategies.

## Conclusion

The current study begins to address to the paucity of evidence regarding the collection of resource-use data in the rare genetic disease population by developing, contextualising, translating, and validating a new instrument, the CSRI-Ra. The availability of this sufficiently standardised tool allows estimation of economic impacts, and a better understanding of the service and resource utilisation patterns surrounding rare genetic diseases in Hong Kong. This is important for near-term and long-term monitoring of the resource consequences of rare genetic diseases. This CSRI-Ra would have value as a standard resource-use questionnaire in the rare genetic disease population, and provides a tool for use in economic evaluations in the future, thereby helping to inform planning for efficient and effective healthcare. The adaptation of the CSRI-Ra to other populations and healthcare systems may facilitate international research and provide a better understanding of the cost of rare genetic diseases.

## Supplementary Information

Supplementary Information 1.

## Data Availability

The CSRI-Ra, CSRI-Ra manual, study protocol, informed consent form, and datasets generated during and/or analysed during the current study are available from the corresponding authors on reasonable request.
