# Supplementary material for: Client Service Receipt Inventory as a standardised tool for measurement of socio-economic costs in the rare genetic disease population (CSRI-Ra)
Source: Sci Rep. 2021 Dec 13;11:23837. doi: 10.1038/s41598-021-03379-5 (PMC8668911; doi:10.1038/s41598-021-03379-5)
Supplement: Supplementary file 1 — Supplementary Information 1. [file 41598_2021_3379_MOESM1_ESM.docx]

**Supplementary document**

***Translation and validation of CSRI-Ra***

Through forward and backward translations, the baseline translated versions of the CSRI-Ra had been developed. The content and language of the English and Traditional Chinese versions of the CSRI-Ra were addressed through email and telephone communication with all focus group participants, face-to-face discussion with ten bilingual university students, and more formally through face-to-face interviews with one senior nurse and two medical social workers.

A number of comments were generated that involved country-specific suggestions and language refining for enhancing the understanding, definition, or measurement of the CSRI-Ra. A particular area of discussion concerned the demographic categorisations that match the standard in Hong Kong context, thereby making it easier to compare the patient groups with the general population. For example, in relation to ethnicity, according to the Hong Kong Census data, 94% of the people in Hong Kong are Chinese (ethnically speaking, Han Chinese).(1) The remaining 6% of the population consists of a variety of ethnicities, with the majority being “Filipino” and “Indonesian”. Ethnicities under “others” represent less than 0.05% of the Hong Kong population.(1) In the CSRI-Ra, participants with ethnicity other than “Chinese” can select “others” and specify rather than keeping a list of choices. This also helps to shorten the length of the tool.

A second set of comments revolved around the definition of the terminologies. It was discussed that the definition or explanation of some terminologies, such as audiologist, should be provided to assist the participants’ understanding. Since this is a self-completed/proxy-completed tool, sufficient information should be given for a lay person to understand. With reference to previous CSRIs, a manual was prepared which contained explanatory notes for particular questions or items.

The CSRI-Ra was further revised in its original English version and in the translated Chinese version, in the light of recommendations on content, terminology and language. Upon further discussing and reviewing of the tool, the tool’s face validity and semantic equivalence was achieved.

Alternate-form reliability between English and Chinese versions

A total of eight bilingual patients (n=4) and carers (n=4) completed both the English and Chinese versions of the CSRI-Ra. A mean time gap between the completion of the English and Chinese versions was found to be 51.4 days. The overall ICC was excellent being 0.91 (95% CI 0.89 – 0.92). The ICC for the patient’s (self-completed) and carer’s (proxy-completed) versions were between good and excellent (ICC 0.89; 95% CI 0.86 – 0.92), and excellent (ICC 0.93; 95% CI 0.91 – 0.95), respectively.

Reference:

1. Census and Statistics Department, Hong Kong Special Administrative Region. 2016 Population By-census. Thematic Report: Ethnic Minorities. <https://www.statistics.gov.hk/pub/B11201002016XXXXB0100.pdf> (2017).
